# Effects of acepromazine, xylazine and propofol on spinal reflexes in healthy dogs

**DOI:** 10.1002/vms3.70009

**Published:** 2024-09-24

**Authors:** Ali Sheykhbahaedinzade, Ali Asghar Sarchahi, Hossein Kazemi Mehrjerdi

**Affiliations:** ^1^ Graduated from Faculty of Veterinary Medicine Ferdowsi University of Mashhad Mashhad Iran; ^2^ Department of Clinical Sciences Faculty of Veterinary Medicine Ferdowsi University of Mashhad Mashhad Iran

**Keywords:** acepromazine, neurological examinations, propofol, spinal reflexes, xylazine

## Abstract

**Background:**

In the neurological examination, it is crucial to identify the possible location of the lesion in order to determine the appropriate treatment process. In aggressive animals, chemical restraint may be necessary due to their non‐cooperative behaviour. However, sedatives may distort the results of examinations. Therefore, a drug should be found that has minimal impact on the examination results.

**Objectives:**

To investigate the effects of acepromazine, xylazine, and propofol on spinal reflexes in healthy dogs.

**Methods:**

In a randomized, blinded study, ten native adult mixed‐breed dogs were participated in three groups with a 1‐week washout period between each group. Before performing each step, the spinal reflexes were evaluated. Then, in the first group, acepromazine (0.05 mg/kg, IM), in the second group, xylazine (1 mg/kg, IM), and in the third group, propofol (3 mg/kg, IV for initial bolus and 0.1 mg/kg/min for maintenance) were injected for sedation. The spinal reflexes were reevaluated at maximum sedation and at 15, 30, and 45 min thereafter.

**Results:**

Acepromazine increased the patellar reflex and decreased the panniculus reflex. Xylazine increased the cranial tibial reflex and decreased the panniculus reflex, while propofol decreased the withdrawal, and extensor carpi radialis reflexes, and suppressed the palpebral and gag reflexes.

**Conclusions:**

The drugs used in the present study did not have a significant impact on the most important reflexes evaluated in neurological examinations. Among the drugs, acepromazine has the least effects compared to other drugs, making it a suitable choice for sedation.

## Graphical abstract

This study investigated the effects of acepromazine, xylazine, and propofol on spinal reflexes in healthy dogs. Ten mixed‐breed dogs were sedated with each drug in a randomized, blinded design. Spinal reflexes were evaluated before sedation, at maximum sedation, and at 15, 30, and 45 min thereafter. Acepromazine increased patellar reflex and decreased panniculus reflex. Xylazine increased cranial tibial reflex and decreased panniculus reflex. Propofol decreased withdrawal and extensor carpi radialis reflexes, and suppressed palpebral and gag reflexes. Acepromazine showed the least specific effects on spinal reflexes, suggesting its potential suitability for sedating aggressive dogs during neurological examinations. Further research is needed to optimize protocols for safe and effective sedation in neurological examinations.

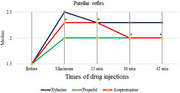

## 1 INTRODUCTION

For accurate lesion localization in the nervous system, neurological examinations can be used, which are non‐invasive and cost‐effective evaluation methods (DeLahunta et al., 2021). Functional disorders of the nervous system can be caused by various factors (Bongartz et al., 2020). Sometimes the symptoms of neurological involvement are the secondary effects of the disease in other organs, which can be distinguished through these examinations. After determining the precise location of damage in one or more areas, we can provide differential diagnoses based on our history and physical examination, ensuring the accuracy of our diagnosis (Jeffery, 2001).

In the clinical setting, patients are typically evaluated in the lateral recumbent position to facilitate the observation of spinal reflexes during neurological examinations (DeLahunta et al., 2021). However, in some cases, the examination may require sedation, especially if the patient is experiencing anxiety or pain (Grimm et al., 2015). During physical restraint, patients may exhibit increased tension or excitement, which can prevent the accurate evaluation of reflexes. In addition, the physical restraint of patients with spinal cord injuries carries the risk of causing additional damage (Horsley et al., 2021). Therefore, the use of chemical restraints becomes necessary. However, it is important to note that chemical drugs may affect spinal reflexes and potentially change the outcomes of neurological examinations (Horsley et al., 2021). Among the drugs used as sedatives in veterinary medicine are acepromazine, xylazine and propofol (Grimm et al., 2015).

Acepromazine is a common phenothiazine sedative used to reduce excitement and stress during various veterinary procedures (Schneiders et al., 2012). This drug induces relaxation by reducing the activity of the reticular activating system and exerting anti‐dopaminergic effects on the central nervous system (Boothe, 2011).

Xylazine is an alpha 2‐adrenoceptor agonist (Clarke & Trim, 2013). Alpha‐2‐adrenergic agonists are commonly used in veterinary medicine because, apart from their analgesic effect, they offer reliable sedation. The sedative effects of alpha‐2 agonists are mediated by presynaptic binding to alpha‐2‐adrenergic receptors in the upper part of the cerebellum (locus coeruleus). Activation of these presynaptic receptors results in a reduction in the synaptic release of norepinephrine (NE). Norepinephrine is the primary neurotransmitter of the sympathetic nervous system, and a decrease in its levels leads to decreased neurotransmission, resulting in diminished consciousness. In the spinal cord, the analgesic effects of alpha‐2 agonists are caused by reducing the release of NE and substance P from the dorsal horn of the spinal cord (Riviere & Papich, 2018).

Propofol (2,6‐diisopropylphenol) is a potent intravenous narcotic drug and an agonist of gamma‐aminobutyric acid (GABA) receptors. Its fast and uniform action without stimulation, relatively short half‐life, rapid elimination, and low probability of postoperative nausea and vomiting make it a versatile hypnotic drug. It is commonly used for sedation and anaesthesia during surgery (Sahinovic et al., 2018).

Horsley et al. (2021) conducted a study on the impact of sedative drugs on the patellar and withdrawal reflexes. They reported that certain sedative drugs can actually increase spinal reflexes. Saberfard et al. (2022) published a study on the effects of sedatives and anaesthetics on spinal reflexes in healthy dogs. In these studies, contrary to the expectation that these drugs would reduce reflexes, they had no effect on some reflexes or even increased them. Despite these studies, there remains a significant gap in our understanding of how different sedatives impact neurological examinations. Our study aims to address this gap by investigating the effects of three commonly used sedative drugs—acepromazine, xylazine, and propofol—on spinal reflexes in healthy dogs. In the study conducted by Saberfard et al. (2022), an anaesthetic dose of propofol was administered, which caused the loss of all reflexes. This rendered it unsuitable for neurological examination, as the preservation of reflexes is crucial for assessing neurological function (Saberfard et al., 2022). In contrast, we focused on finding suitable sedation protocols that would allow for effective neurological assessment while maintaining necessary reflexes. By comparing these drugs at sedative doses, we aim to identify options that provide adequate sedation with minimal interference on spinal reflexes, thereby improving the accuracy and reliability of neurological examinations in sedated dogs. This research is crucial for developing evidence‐based sedation protocols for neurological examinations, particularly in cases where chemical restraint is necessary due to patient anxiety, pain, or risk of further injury.

## 2 MATERIALS AND METHODS

The study protocol was assessed by the Research Committee of the Faculty of Veterinary Medicine and approved by the Research Ethics Committee of Ferdowsi University of Mashhad, Mashhad, Iran (Approval ID: IR.UM.REC.1401.179). The present study was conducted on 10 adult mixed‐breed dogs with an average age of 23.8 ± 3.64 months (ranging from 12 to 48 months) and an average weight of 22.60 ± 1.30 kg (ranging from 16 to 28 kg), all in healthy clinical conditions. To ensure all participating dogs were healthy, we implemented a multi‐step evaluation process. First, a board‐certified Small Animal Internal Medicine specialist (AAS) performed a complete physical examination, including vital signs, body condition, and examination of all major body systems. Additionally, a cardiac assessment looked for murmurs, arrhythmias, and employed ECG if needed. Finally, a behavioural evaluation ensured the dogs were comfortable with handling and wouldn't experience undue stress during the study. Dogs with any underlying medical conditions or receiving concurrent medications, presence of systemic or diagnosed diseases, cardiac abnormalities (e.g., heart murmurs, arrhythmias), temperamental issues (e.g., aggression, fearfulness, stubbornness), recent surgical procedures or illnesses, high levels of anxiety or fear that could potentially induce undue stress during testing and affect the results, as well as dogs with orthopaedic or neurological problems, were excluded from the study. The dogs were transferred from a dog shelter to the teaching hospital of the Faculty of Veterinary Medicine with the written consent of their owner. After completing the project, they were returned to their original location. All the dogs were participated in three treatment groups, with a 1‐week interval between each group. In the first group, acepromazine (Neurotranq, Alfasan, Woerden, *Holland*) was administered intramuscularly at a dose of 0.05 mg/kg (Grimm et al., 2015), in the second group, xylazine hydrochloride (*Alfasan*, Woerden, Holland) was administered intramuscularly at a dose of 1 mg/kg (Ambrisko & Hikasa, 2002) and in the third group, propofol (Lipuro 10 mg/ml, B. Braun Melsungen AG, Melsungen, Germany) was administered for sedation at a dose of 3 mg/kg by a slow intravenous injection (within 60 seconds) for induction, followed by a maintenance of 0.1 mg/kg/min (Grimm et al., 2015). Spinal reflexes were measured before the administration of the drug, at the peak of sedation, and at 15, 30, and 45 min afterward. The maximum level of sedation was defined as not responding to environmental stimuli (such as touch), lying on the side, or being unable to hold the head (Horsley et al., 2021). To assess the effects of drugs from the second and third groups, the dogs were given a week of rest before the next drug was tested. All examinations were conducted by a single individual who was blinded to the treatment groups. This examiner was unaware of which specific drug had been administered to each dog and did not participate any aspect of drug preparation or administration. This blinding process was implemented to minimize potential bias in the assessment of neurological reflexes across the different treatment groups.

### 2.1 Classification of reflexes

The spinal reflexes were assessed by positioning the animals in right lateral recumbency and eliciting the reflexes on the left limb. After evaluation, each reflex was graded and recorded as follows: absent (0), reduced (1+), normal (2+), increased (3+), and clonic (4+) (DeLahunta et al., 2021). The order of the reflexes was as follows: patellar reflex, cranial tibial reflex, extensor carpi radialis reflex, pelvic limb withdrawal reflex, thoracic limb withdrawal reflex, cutaneous trunci (panniculus) reflex, perineal reflex, palpebral reflex, and gag reflex.

To assess the patellar reflex, the pelvic limb was positioned in a partially flexed position, and the patellar ligament was struck with a patellar hammer. The response is a brisk extension of the stifle.

The cranial tibial reflex was evaluated by striking the cranial tibial muscle belly immediately distal to the proximal end of the tibia with the relaxed limb and slight extension of the hock. The normal response is flexion of the hock.

The extensor carpi radialis reflex was assessed by striking the muscle belly of the extensor carpi radialis immediately distal to the elbow joint with the relaxed limb and carpal flexion. The normal response is extension of the carpus.

Thoracic and pelvic limb withdrawal reflexes were evaluated by compressing the skin of the fourth digits of the forelimb and rear limb respectively, using a pair of tissue forceps. A normal response is the flexion of the entire limb.

For assessing the cutaneous trunci (panniculus) reflex, the skin on both sides of the spine was stimulated in the lumbar region. The normal response is the contraction of the cutaneous trunci muscle on both sides, which is evident by the movement of the skin over the thorax.

To elicit the perineal reflex, the perineum was stimulated with a pair of tissue forceps. The normal response is the contraction of the anal sphincter and pulling down of the tail.

The palpebral reflex was performed by gently touching the corners of the eyelids. The immediate and complete closing of the eyelids is the normal response to this reflex.

The gag reflex was evaluated by applying external pressure to the hyoid region. Swallowing is a normal response in a healthy animal.

### 2.2 Statistical analysis

The reflexes measured at different timepoints in each drug group were compared using Friedman's non‐parametric statistical method. If the results were found to be significant, Wilcoxon's method was used for further comparison. All analyses were performed using SPSS 24 software (IBM, Armonk, New York), and the significance level was set at *p* < 0.05.

## 3 RESULTS

**FIGURE 1:**
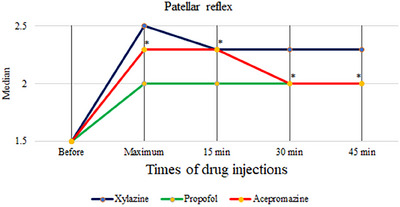
Patellar reflex response after injection of treatment agents in 10 healthy dogs. * indicates significant increase (*P*<0.05) in patellar reflex between baseline and post‐drug administration of acepromazine. Xylazine and propofol did not significantly change the reflex.

The median and percentiles of the scores obtained in the treatment groups in the present study are presented in Tables 1, 2, 3. The main observed effects of the drugs investigated in this study on spinal reflexes are summarized in Table 4. The results showed that in the acepromazine group, the median score of patellar reflex increased significantly at all timepoints after drug injection compared to before drug injection (*p* = 0.001) (Figure 1), while in the xylazine and propofol groups, the increase in the median patellar reflex was not statistically significant (*p* = 0.434 and *p* = 0.741, respectively).

**TABLE 1:** Median and percentiles (25th and 75th) of spinal reflex scores after acepromazine injection in 10 healthy dogs.

Drug	Reflex	Evaluation time	N	Percentiles		
				25th	50th (Median)	75th
Acepromazine	Patellar	before	10	1.0	1.5	2.0
		maxsed	10	1.5	2.3	2.6
		drug15	10	2.0	2.3	2.6
		drug30	10	2.0	2.0	3.0
		drug45	10	1.9	2.0	2.6
	cranial tibial	before	10	1.5	2.0	2.1
		maxsed	10	2.0	2.0	2.5
		drug15	10	2.0	2.0	2.5
		drug30	10	2.0	2.0	2.5
		drug45	10	2.0	2.0	2.5
	extensor carpi radialis	before	10	1.0	1.3	1.6
		maxsed	10	0.9	1.5	1.6
		drug15	10	1.4	1.5	2.0
		drug30	10	1.0	1.5	1.5
		drug45	10	1.4	1.5	1.6
	thoracic limb withdrawal	before	10	1.9	2.0	2.0
		maxsed	10	1.8	2.0	2.0
		drug15	10	1.9	2.0	2.0
		drug30	10	1.8	2.0	2.3
		drug45	10	1.8	2.0	3.0
	pelvic limb withdrawal	before	10	1.8	2.0	2.1
		maxsed	10	1.0	1.8	2.3
		drug15	10	1.4	2.0	2.3
		drug30	10	1.4	1.8	2.3
		drug45	10	1.5	2.0	2.0

**TABLE 2:** Median and percentiles (25th and 75th) of spinal reflex scores after xylazine injection in 10 healthy dogs.

Drug	Reflex	Evaluation time	N	Percentiles		
				25th	50th (Median)	75th
Xylazine	Patellar	before	10	0.9	1.5	3.0
		maxsed	10	2.0	2.5	2.6
		drug15	10	2.0	2.3	2.6
		drug30	10	2.0	2.3	2.5
		drug45	10	2.0	2.3	2.6
	cranial tibial	before	10	1.0	2.0	2.0
		maxsed	10	2.0	2.0	2.5
		drug15	10	1.5	2.0	2.1
		drug30	10	2.0	2.0	2.1
		drug45	10	2.0	2.0	2.6
	extensor carpi radialis	before	10	1.0	1.3	2.0
		maxsed	10	1.0	1.3	1.5
		drug15	10	1.0	1.5	1.6
		drug30	10	1.0	1.5	2.0
		drug45	10	1.0	1.3	2.0
	thoracic limb withdrawal	before	10	1.0	2.0	3.0
		maxsed	10	0.5	1.3	2.1
		drug15	10	0.5	1.5	2.0
		drug30	10	0.9	1.0	2.0
		drug45	10	1.0	2.0	2.0
	pelvic limb withdrawal	before	10	1.3	2.0	2.8
		maxsed	10	0.9	1.3	2.6
		drug15	10	0.9	1.0	3.0
		drug30	10	1.0	1.3	2.6
		drug45	10	0.9	1.5	2.1

**TABLE 3:** Median and percentiles (25th and 75th) of spinal reflex scores after propofol injection in 10 healthy dogs.

Drug	Reflex	Evaluation time	N	Percentiles		
				25th	50th (Median)	75th
Propofol	Patellar	before	10	0.9	1.5	3.0
		maxsed	10	1.4	2.0	2.3
		drug15	10	1.3	2.0	3.0
		drug30	10	1.5	2.0	2.6
		drug45	10	1.9	2.0	2.6
	cranial tibial	before	10	1.0	2.0	2.0
		maxsed	10	2.0	2.0	2.0
		drug15	10	1.9	2.0	2.0
		drug30	10	1.5	1.8	2.1
		drug45	10	1.5	2.0	2.5
	extensor carpi radialis	before	10	1.0	1.5	2.0
		maxsed	10	1.0	1.5	1.8
		drug15	10	1.0	1.3	1.5
		drug30	10	1.0	1.0	1.1
		drug45	10	1.0	1.0	1.5
	thoracic limb withdrawal	before	10	1.5	2.0	2.6
		maxsed	10	1.0	1.5	2.0
		drug15	10	1.4	2.0	2.0
		drug30	10	1.4	2.0	2.0
		drug45	10	1.0	2.0	2.0
	pelvic limb withdrawal	before	10	2.0	2.0	3.3
		maxsed	10	1.0	1.3	2.0
		drug15	10	1.0	2.0	2.0
		drug30	10	1.8	2.0	2.0
		drug45	10	1.9	2.0	2.0

**TABLE 4:** Major observed effects of drugs investigated in this study on spinal reflexes.

Reflex Group	Patellar	Cranial tibial	Extensor carpi radialis	Pelvic limb withdrawal	Thoracic limb withdrawal	cutaneous trunci (panniculus)	Perineal	Palpebral	Gag
Acepromazine	↑	—	—	—	—	↓	—	↓	↓
Xylazine	—	↑	—	—	—	↓	—	↓	↓
Propofol	—	—	↓	↓	—	—	—	↓	↓

↑In most of the timepoints, there was a significant increase in the reflex compared to the timepoint before drug injection.

↓In most of the timepoints, there was a significant reduction in the reflex compared to the timepoint before drug injection.

—In most of the time points, the reflex did not change significantly compared to the timepoint before drug injection.

The median score of cranial tibial reflex did not change significantly after the administration of acepromazine (*p* = 0.253) and propofol (*p* = 0.660) but exhibited a significant increase in the xylazine group at maximum sedation (*p* = 0.015), 30 min (*p* = 0.046) and 45 min (*p* = 0.024), except for the 15th minute, which was not significant compared to before the drug injection (*p* = 0.163).

The median score of the extensor carpi radialis reflex remained unchanged in the acepromazine and xylazine groups (*p* = 0.137 and *p* = 0.967, respectively) but decreased significantly in the propofol group at 30 min (*p* = 0.024) and 45 min (*p* = 0.023).

**FIGURE 2:**
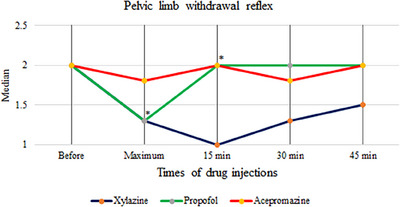
Pelvic limb withdrawal reflex response after injection of treatment agents in 10 healthy dogs. * indicates significant decrease (p<0.05) in pelvic limb withdrawal reflex between baseline and post‐drug administration of propofol. Acepromazine and xylazine did not significantly change the reflex.

The median score of pelvic limb withdrawal reflex showed no significant changes in the acepromazine and xylazine groups (*p* = 0.406 and *p* = 0.060, respectively) but decreased significantly in the propofol group at maximum sedation (*p* = 0.016) and up to 15 min later (*p* = 0.031) (Figure 2).

The median score of the thoracic limb withdrawal reflex did not change significantly in any group (acepromazine: *p* = 0.920, xylazine: *p* = 0.163, propofol: *p* = 0.099).

The median score of the cutaneous trunci (panniculus) reflex decreased significantly at all timepoints following the injection of acepromazine (*p* = 0.001) and xylazine (*p* < 0.001) but not after propofol administration (*p* = 0.258).

The median perineal reflex did not show any significant change in any group (acepromazine: *p* = 0.487, xylazine: *p* = 0.158, propofol: *p* = 0.168). However, the median of palpebral and gag reflexes showed a significant decrease at all timepoints compared to before injection in all three groups (acepromazine: *p* = 0.003, xylazine: *p* = 0.005, propofol: *p* < 0.001 for palpebral and acepromazine: *p* = 0.002, xylazine: *p* < 0.001, propofol: *p* < 0.004 for gag).

## 4 DISCUSSION

The present study investigated the effects of acepromazine, xylazine, and propofol on spinal reflexes in dogs. The goal was to identify a drug suitable for sedating dogs with spinal cord injuries without affecting neurological examinations.

The unit of nerve function is the reflex arc. Reflexes that involve only one synapse in their arc are called monosynaptic. Examples of these include the patellar reflex, cranial tibial reflex, and extensor carpi radialis reflex. On the other hand, reflexes that involve more intermediate neurons in their message transmission path are called polysynaptic. Examples of these include the thoracic and pelvic limb withdrawal reflexes, as well as the panniculus and perineal reflexes (Horsley et al., 2021). Among these reflexes, the patellar and the withdrawal reflexes are the most important reflexes typically examined in neurological assessments (DeLahunta et al., 2021).

In the present study, our primary focus was on assessing neurological reflexes under conditions that mirror routine clinical examinations. While most dogs were cooperative, some cases required minimal physical restraint, reflecting real‐world clinical scenarios. Importantly, we were able to examine all spinal reflexes in each case, regardless of the dog's behaviour. This approach allowed us to evaluate the drugs' effects on neurological findings in a practical, clinical context, which was the main objective of our study. We did not formally assess sedation levels or examination feasibility, as these were outside the scope of our research design.

In the present study, acepromazine increased the patellar reflex but had no significant effect on other reflexes commonly assessed during neurological examinations, such as the cranial tibial, extensor carpi radialis, and thoracic and pelvic limb withdrawal reflexes. This is in contrast to previous studies reporting a decrease in the patellar reflex with chlorpromazine (Hudson & Domino, 1963; Keary & Maxwell, 1967). Although the exact cause of these differences is not yet clear, potential contributing factors could include variations in medication dosage, species differences, or the specific reflex assessment techniques used in each study. Acepromazine, as a muscle relaxant, exerts its effects by binding to dopamine receptors in various parts of the brain including the basal nuclei, hypothalamus, limbic system, brainstem and reticular activating system. This binding suppresses the central nervous system, leading to sedation and decreased motor activities (Hernández‐Godínez et al., 2019). Thus, acepromazine can increase the descending inhibitory effect of these areas on the spinal cord and enhance the patellar reflex by acting on these areas (Engberg et al., 1968). On the other hand, acepromazine seems to enhance the visibility of the patellar reflex because some dogs tense up so much that the reflex becomes less noticeable (Horsley et al., 2021; Saberfard et al., 2022) (Video clip 1). Consistent with Bergadano et al. (2009) our findings showed no change in withdrawal reflexes suggesting that acepromazine can be used without disturbing the results of the examination of these reflexes (Bergadano et al., 2009). The reduction of gag and palpebral reflexes aligns with acepromazine's central nervous system suppression (Horsley et al., 2021).

While a definitive conclusion cannot be drawn yet, the lack of effect of acepromazine on most reflexes suggests its potential use for aggressive dogs without compromising examination results. Further research with variable doses and intervals in healthy and neurologically affected dogs is necessary to determine optimal administration methods.

In the present study, xylazine increased the cranial tibial reflex and had no effect on the patellar and extensor carpi radialis reflexes (Table 4). Furthermore, it had no significant effect on polysynaptic reflexes, except for the panniculus, which exhibited a decrease response. Additionally, xylazine reduced gag and palpebral reflexes. Our findings regarding the cranial tibial reflex are partially consistent with those of Saberfard et al. (2022) who reported increased patellar and cranial tibial reflexes, but no change in thoracic and pelvic limb withdrawal reflexes, following medetomidine administration. This partial concordance can be explained by the shared alpha‐2 adrenergic agonist properties of both xylazine and medetomidine (Clarke & Trim, 2013). The increase in the cranial tibial reflex observed in our study can be attributed to the muscle relaxant effect of xylazine, as suggested by Saberfard et al. (2022) for medetomidine. They proposed that sedation induced by these drugs enhances the visibility of reflexes by preventing muscle contractions that may occur due to extreme stress in unsedated animals. However, unlike Saberfard et al. (2022), xylazine did not affect the patellar reflex. This discrepancy might be attributed to two factors. Firstly, medetomidine might have a more pronounced effect on muscle relaxation compared to xylazine. Secondly, the dose of xylazine used in our study (1 mg/kg) might have been insufficient to induce a similar effect on the patellar reflex, considering the typical sedation dosage range of 1–3 mg/kg (Tyner et al., 1997).

In the current study, propofol decreased the extensor carpi radialis (a monosynaptic reflex). This finding is consistent with previous research suggesting that propofol has a weaker inhibitory effect on monosynaptic reflexes compared to polysynaptic reflexes (Baars et al., 2009; Matute et al., 2004). Propofol decreased the pelvic limb withdrawal reflex (a polysynaptic reflex) in our study. This observation aligns with the findings of Baars et al. (2009) who reported a stronger inhibitory effect of propofol on the RIII reflex, a polysynaptic nociceptive withdrawal reflex (Baars et al., 2009). The inhibitory effects of propofol on polysynaptic reflexes, such as withdrawal reflexes, are proposed to occur through its action on the GABA receptor, opening Cl channels, hyperpolarizing the postsynaptic cell membrane, and inhibiting postsynaptic neurons (Stegmann & Bester, 2001). Additionally, Matute et al. (2004) demonstrated that propofol can inhibit non‐pain reflexes by influencing the motor nerves of the ventral root of the spinal cord (Matute et al., 2004). In our study, propofol reduced palpebral and gag reflexes. The Gag reflex is a complex response involving multiple cranial nerves (5th, 9th, and 10^th^) and is highly sensitive to centrally depressing drugs (Khojasteh & Vesal, 2023). This finding is consistent with previous research suggesting that propofol can inhibit pharyngolaryngeal activity and reduce the resting tone threshold of the upper oesophageal sphincter, even at non‐anaesthetic doses (Gemma et al., 2016). Saberfard et al. (2022) found that general anaesthesia with propofol eliminated all spinal reflexes, concluding that anaesthetic doses of propofol are not suitable for neurological examination in dogs. In contrast, our study used a lower sedative dose of propofol (3 mg/kg), which allowed for neurological examination without affecting most important reflexes, such as the patellar and thoracic limb withdrawal reflexes. However, this dosage of the drug reduced some reflexes, so further studies are needed to determine an appropriate dosage of the drug that can provide the necessary sedation without affecting the reflexes.

One of the limitations of this study was the small number of dogs participating in each drug group. This may affect the obtained results. With this study, we tried to use dogs of similar age and weight to minimize excessive data dispersion. This is a preliminary study, which, in our opinion, yielded favourable results. Therefore, it could be further investigated in a large sample of dogs in the future.

Another limitation of this study was that the amount of force applied by the hand to check the reflex may vary. To minimize this effect, all reflex tests were conducted by a single researcher at a consistent location on the limb.

It is also important to note that this study was conducted in healthy animals, and its results may vary in animals with neurological diseases.

## 5 CONCLUSION

Our study reveals that acepromazine, xylazine, and propofol had minimal impact on most reflexes crucial for neurological examinations in dogs, with acepromazine showing the least specific effects on spinal reflexes. These findings suggest acepromazine as a potential suitable option for sedating aggressive dogs during neurological examinations, minimizing physiological disturbances. However, further research is needed to determine optimal dosages, evaluate effects in dogs with neurological conditions, and assess the impact of drug combinations on reflex responses, ultimately refining protocols for safe and effective sedation during neurological examinations of aggressive or anxious dogs.

## AUTHOR CONTRIBUTIONS

Conceptualization: **Ali Asghar Sarchahi**. Methodology: **Ali Asghar Sarchahi; Ali Sheykhbahaedinzade**; **Hossein Kazemi Mehrjerdi**. Writing‐original draft preparation: **Ali Asghar Sarchahi**. Writing‐review and editing: **Ali Asghar Sarchahi; Ali Sheykhbahaedinzade; Hossein Kazemi Mehrjerdi**. Supervision: **Ali Asghar Sarchahi**.

## Funding information

Research Council of Ferdowsi University of Mashhad, Grant No. 58986

## CONFLICT OF INTEREST STATEMENT

The authors have no conflicts of interest to declare.

## ETHICS APPROVAL

The study protocol was assessed by the Research Committee of the Faculty of Veterinary Medicine and approved by the Research Ethics Committee of Ferdowsi University of Mashhad, Mashhad, Iran (Approval ID: IR.UM.REC.1401.179).

## Supporting information



Supporting Information: Video clip 1: Comparison of the patellar reflex before and after the administration of acepromazine. The increased visibility of the patellar reflex after the administration of acepromazine is quite evident.

## Data Availability

The datasets used and analysed in this study are available from the corresponding author upon reasonable request.

## References

[vms370009-bib-0001] Ambrisko, T. , & Hikasa, Y. (2002). Neurohormonal and metabolic effects of medetomidine compared with xylazine in beagle dogs. Canadian Journal of Veterinary Research, 66, 42.. 11858648 PMC226981

[vms370009-bib-0002] Baars, J. H. , Mager, R. , Dankert, K. , Hackbarth, M. , von Dincklage, F. , & Rehberg, B. (2009). Effects of sevoflurane and propofol on the nociceptive withdrawal reflex and on the H reflex. The Journal of the American Society of Anesthesiologists, 111, 72–81. 10.1097/ALN.0b013e3181a4c706 19512883

[vms370009-bib-0003] Bergadano, A. , Andersen, O. K. , Arendt‐Nielsen, L. , Theurillat, R. , Thormann, W. , & Spadavecchia, C. (2009). Plasma levels of a low‐dose constant‐rate‐infusion of ketamine and its effect on single and repeated nociceptive stimuli in conscious dogs. The Veterinary Journal, 182, 252–260.. 18706837 10.1016/j.tvjl.2008.06.003

[vms370009-bib-0004] Bongartz, U. , Nessler, J. , Maiolini, A. , Stein, V. M. , Tipold, A. , & Bathen‐Nöthen, A. (2020). Vestibular disease in dogs: Association between neurological examination, MRI lesion localisation and outcome. Journal of Small Animal Practice, 61, 57–63.. 31515806 10.1111/jsap.13070

[vms370009-bib-0005] Boothe, D. M. (2011). Small animal clinical pharmacology and therapeutics. Elsevier Health Sciences.

[vms370009-bib-0006] Clarke, K. W. , & Trim, C. M. (2013). Veterinary anaesthesia e‐book. Elsevier Health Sciences.

[vms370009-bib-0007] DeLahunta, A. , Glass, E. , & Kent, M. (2021). de Lahunta's veterinary neuroanatomy and clinical neurology. Elsevier Health Sciences.

[vms370009-bib-0008] Engberg, I. , Lundberg, A. , & Ryall, R. (1968). Reticulospinal inhibition of transmission in reflex pathways. The Journal of Physiology, 194, 201–223.. 4295754 10.1113/jphysiol.1968.sp008402 PMC1365682

[vms370009-bib-0009] Gemma, M. , Pasin, L. , Oriani, A. , Agostoni, M. , Palonta, F. , Ramella, B. , Bussi, M. , & Beretta, L. (2016). Swallowing impairment during propofol target‐controlled infusion. Anesthesia and Analgesia, 122, 48–54.. 26049781 10.1213/ANE.0000000000000796

[vms370009-bib-0010] Grimm, K. A. , Lamont, L. A. , Tranquilli, W. J. , Greene, S. A. , & Robertson, S. A. (2015). Lumb and Jones Veterinary Anesthesia and Analgesia. John Wiley & Sons, Inc.

[vms370009-bib-0011] Hernández‐Godínez, B. , Bonilla Jaime, H. , Poblano, A. , Arteaga‐Silva, M. , Medina Hernández, A. , Contreras‐Uribe, A. , & Ibáñez‐Contreras, A. (2019). Effect of different anesthetic mixtures—ketamine‐xylazine, ketamine‐acepromazine and tiletamine‐zolazepam—on the physiological and blood biochemistry parameters of male rhesus monkeys (Macaca mulatta) at different ages. Animal Models and Experimental Medicine, 2, 83–97. 31392301 10.1002/ame2.12062 PMC6600652

[vms370009-bib-0012] Horsley, K. T. , Olby, N. J. , Mitchell, M. A. , Aulakh, K. S. , & Gines, J. A. (2021). Effect of sedation on the neurological examination of the patellar and withdrawal reflexes in healthy dogs. Frontiers in Veterinary Science, 8, 664150. 34041291 10.3389/fvets.2021.664150 PMC8143191

[vms370009-bib-0013] Hudson, R. D. , & Domino, E. F. (1963). Effects of chlorpromazine on some motor reflexes. International Journal of Neuropharmacology, 2, 143–162.

[vms370009-bib-0014] Jeffery, N. (2001). Neurological examination of dogs 1. Techniques In Practice, 23, 118–130.

[vms370009-bib-0015] Keary, E. M. , & Maxwell, D. (1967). A comparison of the effects of chlorpromazine and some related phenothiazines in reducing the rigidity of the decerebrate cat and in some other central actions. British Journal of Pharmacology and Chemotherapy, 30, 400. 6036418 10.1111/j.1476-5381.1967.tb02147.x PMC1557266

[vms370009-bib-0016] Khojasteh, K. , & Vesal, N. (2023). Comparison of propofol infusion rate required to abolish swallowing or pedal withdrawal reflexes in dogs. Veterinary Research Forum, Faculty of Veterinary Medicine. Urmia University. 10.30466/vrf.2021.537306.3220 PMC10003597 36909683

[vms370009-bib-0017] Matute, E. , Rivera‐Arconada, I. , & Lopez‐Garcia, J. (2004). Effects of propofol and sevoflurane on the excitability of rat spinal motoneurones and nociceptive reflexes in vitro. British Journal of Anaesthesia, 93, 422–427. 15277303 10.1093/bja/aeh217

[vms370009-bib-0018] Riviere, J. E. , & Papich, M. G. (2018). Veterinary pharmacology and therapeutics. John Wiley & Sons.

[vms370009-bib-0019] Saberfard, D. , Sarchahi, A. A. , & Mehrjerdi, H. K. (2022). Effect of medetomidine, midazolam, ketamine, propofol and isoflurane on spinal reflexes in healthy dogs. Veterinary Medicine and Science, 8, 2351–2359. 36084272 10.1002/vms3.938 PMC9677393

[vms370009-bib-0020] Sahinovic, M. M. , Struys, M. M. , & Absalom, A. R. (2018). Clinical pharmacokinetics and pharmacodynamics of propofol. Clinical Pharmacokinetics, 57, 1539–1558. 30019172 10.1007/s40262-018-0672-3 PMC6267518

[vms370009-bib-0021] Schneiders, F. I. , Noble, G. K. , Boston, R. C. , Dunstan, A. J. , Sillence, M. N. , & McKinney, A. R. (2012). Acepromazine pharmacokinetics: A forensic perspective. The Veterinary Journal, 194, 48–54. 22534188 10.1016/j.tvjl.2012.03.017

[vms370009-bib-0022] Stegmann, G. , & Bester, L. (2001). Some clinical effects of midazolam premedication in propofol‐induced and isoflurane‐maintained anaesthesia in dogs during ovariohysterectomy. Journal of the South African Veterinary Association, 72, 214–216. 12219917 10.4102/jsava.v72i4.655

[vms370009-bib-0023] Tyner, C. L. , Woody, B. J. , Reid, J. S. , Chafetz, E. P. , Lederer, H. A. , Norton, J. F. , Keefe, T. J. , & Jöchle, W. (1997). Multicenter clinical comparison of sedative and analgesic effects of medetomidine and xylazine in dogs. Journal of the American Veterinary Medical Association, 211, 1413–1417. 9394891

